# Editorial for Special Issue on Neurodynamics

**DOI:** 10.1186/2190-8567-3-10

**Published:** 2013-08-14

**Authors:** Stephen Coombes, Yulia Timofeeva

**Affiliations:** 1 Centre for Mathematical Medicine and Biology, School of Mathematical Sciences, University of Nottingham, Nottingham, NG7 2RD, UK; 2 Department of Computer Science and Centre for Complexity Science, University of Warwick, Coventry, CV4 7AL, UK

## Abstract

“Neurodynamics” is an interdisciplinary area of mathematics where dynamical systems theory (deterministic and stochastic) is the primary tool for elucidating the fundamental mechanisms responsible for the behaviour of neural systems (whether biological or synthetic). A meeting on this topic was held at the International Centre for Mathematical Sciences in Edinburgh from March 5–7 in 2012. In this special issue, we have invited seven of the main contributors to this event to expand on their presentations and highlight the use of mathematics in understanding the dynamics of neural systems.

## 1 Editorial

Despite the historical success of mathematics in neuroscience, it is fair to say that this field is in its infancy compared to many other areas of applied mathematics. One area that is more developed than others is “Neurodynamics,” which we loosely define as the application of techniques from dynamical systems theory to models of neurons and neural systems. At the level of the cell, one is often interested in the temporal evolution of gating variables describing the activity of channels (or clusters of channels), as well as the evolution of membrane voltage at the cell body and along axons and dendrites. For a given single cell model, commonly formulated as a set of ordinary differential equations (say of Hodgkin–Huxley style or nonsmooth integrate-and-fire type), it is of interest to understand the qualitative patterns of temporal activity that can be generated (such as periodic, mixed-mode, bursting, chaotic). Techniques such as those from singular perturbation theory and numerical analysis have proven especially useful in this regard. In particular, understanding patterns of spiking activity (the time at which an action potential occurs) are of relevance for neural information processing and coding at both the single cell and network level. For weakly interacting oscillatory systems, one may use other tools from the dynamical systems arsenal, such as the adjoint method for constructing phase response functions, to build and understand network behaviours such as synchrony. At the network level, one is also interested in developing and analysing models for the evolution of macroscopic brain rhythms (in systems that are neither oscillatory or weakly interacting) relevant to cognitive processing (such as the gamma rhythm) and brain diseases (such as epilepsy). The meeting in Edinburgh from March 5–7 in 2012 entitled “Neurodynamics”^a^ was aimed at addressing precisely these topics. We invited seven of the main contributors at this meeting to expand upon their presentations and contribute to this special issue on Neurodynamics.

The paper by Yu et al. [1] studies spike time reliability in the case of a Morris–Lecar model (for two different types of excitability) perturbed by noise and contributes to the community debate on the explanation of spike time reliability in neurons. The paper primarily focuses on the role of the “action” on the reliability, that is the current delivered over some time interval, concluding that a sharp increase in action just before threshold is key to generating reliable spike-evoking epochs (SEEs). In addition, the authors conclude that low average action also contributes to reliable SEEs and that power in Fourier modes of the natural frequency of an oscillator is not always necessary for reliable firing. A single neuron excitable model is also the topic of the paper by Mitry et al. [2], with a focus on firing threshold manifolds and the role of the anaesthetic propofol on *rebound* spiking. Spiking occurs when a given trajectory crosses the firing threshold which, in the context of a multiple time scale system, can be related to canard orbits (acting as separatrices). Geometric singular perturbation theory is used to understand rebound spiking behaviour of the model and how this can be affected by the interaction of the time scale of the inhibition with the spiking dynamics. Understanding the response of single neuron models to pulsatile stimuli is considered next in the paper by Castejón et al. [3]. This work goes beyond the standard approach that typically utilizes only the infinitesimal phase response curve (PRC), and shows how to capture information about the *amplitude* (and not just phase) of response. The amplitude response function is defined according to a coordinate transformation for which the phase advances at a constant speed and the amplitude changes exponentially (related to the isochrons of the periodic orbit). The application of the framework shows very clearly that phase only descriptions can fail to correctly describe the behaviour of forced neural systems. The dynamics of interacting neuronal populations is the topic of the paper by Merrison et al. [4], moving us up through the scales of neurodynamics and away from the details of single neuron models. These authors focus on a new model of the GPe-STN circuit which is believed to play an important role in Parkinsonian conditions. A bifurcation analysis of the two reciprocally connected neuronal populations (one excitatory and one inhibitory) is used to demonstrate the existence of a region of dynamics with bistability between a steady and oscillating state and suggests novel ways to think about basal ganglia dynamics and Parkinsonism. Networks are also the topic of the paper by Timofeeva et al. [5], though here the emphasis is on the spatial extent of single neurons and their interaction through dendro-dendritic gap junctions. It is shown (for the case of passive and quasi-active dendritic dynamics) how a network response function can be constructed using a “sum-over-trips” formalism, and that the position and strength of gap junction coupling can be used to *tune* network response. As in [2], the paper by Fontolan et al. [6] also makes use of singular perturbation theory, albeit this time to gain insights at the network level on the role of theta-gamma brain rhythms in neuroscience, using a fast spiking model coupled to an oscillator functioning in the theta range. Analytical and approximate expressions are derived for the spike timings exploiting the different time scales of the model. The final paper by Goodfellow and Glendinning [7] considers a network of coupled oscillators with differing intrinsic properties (heterogeneity) and shows that simple additive coupling can give rise to intermittent state transitions similar to those observed in epilepsy. This is an important step in the development of a set of mathematical tools for the study of activity in heterogeneous media that can be used in brain modelling. Indeed, it is hard even at a first approximation to view the brain as a homogeneous system. 

Although mathematical work on Neurodynamics has increased in recent years, the study of heterogeneity, noise, delays, and plasticity needs much further attention. A firmer mathematical framework for treating dynamical systems with these attributes will pave the way for a more comprehensive understanding of the dynamic states of biological neural networks, and their role in facilitating natural computation. We look forward to the time when more mathematicians will take up these grand challenges and use the Journal of Mathematical Neuroscience as a forum for the exchange of results and ideas.

## Competing Interests

The authors declare that they have no competing interests.

## Authors’ Contributions

SC and YT contributed equally to this editorial.
